# Research on the Preparation and Spectral Characteristics of Graphene/TMDs Hetero-structures

**DOI:** 10.1186/s11671-020-03439-1

**Published:** 2020-11-25

**Authors:** Tao Han, Hongxia Liu, Shulong Wang, Shupeng Chen, Kun Yang

**Affiliations:** Key Laboratory for Wide-Bandgap Semiconductor Materials and Devices of Education, the School of Microelectronics, Xidian University, Xi’an, 710071 China

**Keywords:** Graphene/WS_2_, Graphene/MoS_2_, Hetero-structures, Raman spectrum, Photoluminescence spectrum

## Abstract

The Van der Waals (vdWs) hetero-structures consist of two-dimensional materials have received extensive attention, which is due to its attractive electrical and optoelectronic properties. In this paper, the high-quality large-size graphene film was first prepared by the chemical vapor deposition (CVD) method; then, graphene film was transferred to SiO_2_/Si substrate; next, the graphene/WS_2_ and graphene/MoS_2_ hetero-structures were prepared by the atmospheric pressure chemical vapor deposition method, which can be achieved by directly growing WS_2_ and MoS_2_ material on graphene/SiO_2_/Si substrate. Finally, the test characterization of graphene/TMDs hetero-structures was performed by AFM, SEM, EDX, Raman and PL spectroscopy to obtain and grasp the morphology and luminescence laws. The test results show that graphene/TMDs vdWs hetero-structures have the very excellent film quality and spectral characteristics. There is the built-in electric field at the interface of graphene/TMDs heterojunction, which can lead to the effective separation of photo-generated electron–hole pairs. Monolayer WS_2_ and MoS_2_ material have the strong broadband absorption capabilities, the photo-generated electrons from WS_2_ can transfer to the underlying *p*-type graphene when graphene/WS_2_ hetero-structures material is exposed to the light, and the remaining holes can induced the light gate effect, which is contrast to the ordinary semiconductor photoconductors. The research on spectral characteristics of graphene/TMDs hetero-structures can pave the way for the application of novel optoelectronic devices.

## Introduction

The size of traditional silicon-based metal oxide semiconductor (CMOS) transistors become smaller with increase in the chip integration, and the preparation processes of device become much more complicated, so researchers have begun to focus on the ultra-thin hetero-structure-based optoelectronics [1, 2]. The two-dimensional (2D) hetero-structures can be combined by the weak van der Waals (vdWs) force between the layers and the strong covalent bond of the layer. The layers can be separated by breaking the weak van der Waals bond and then easily transferred to other substrates [3]. The formation of new atomic-level 2D vdWs hetero-structures can be achieved by stacking the different 2D materials, and the synergistic effects of 2D hetero-structures become very important. Meanwhile, there are charge rearrangements and structural changes between adjacent crystals in hetero-structures, which can be regulated by adjusting the relative orientation of each element material. The different hetero-structures can not only maintain the properties of single material, but also produce the new physical characteristics under the synergy effect [4–6]. The vdWs hetero-structures are the material guarantee for exploring the new physical phenomena and laws, which can provide more possibilities for the nano-electronic devices with excellent photoelectric properties.

Since the 2D-crystalline materials have the strong interactions against a light, they have attracted extensive attentions as photosensitive materials [7]. Graphene is the atomic-level 2D material with excellent electrical, optical and mechanical properties, which has the wide application in the optoelectronics field [8–10]. However, the defect of zero band gap limits the application and development of graphene. The structure of 2D transition metal dichalcogenide (TMDs) materials is similar to that of graphene, and its band gap width changes with the layer number and thickness [11, 12]. The TMDs and graphene materials with complementary advantages are superimposed together, which can promote the application of graphene and TMDs materials in the photoelectric detection field [13–15]. The high mobility of graphene can ensure the rapid response of device, and the Van Hof singularity in electronic state density of TMDs materials ensures the strong interaction between light and materials, which can effectively enhance the absorption of light and the generation of electron–hole pairs [16, 17]. The 2D hetero-structures were widely used in the new electronic and optoelectronic devices, which is due to its transport characteristics of charge tunneling or charge accumulation, flexible energy band engineering and unique interlayer exciton characteristics. Therefore, the interlayer synergy interaction between graphene and TMDs materials can effectively control the band structure, magnetic properties and the exciton properties of hetero-structures. The graphene/TMDs hetero-structures have the high photosensitivity and light response performance, which is due to the strong quantum confinement effect [18, 19]. At present, there are the few studies on the controllable preparation methods of the large-area, large-size and high-quality graphene/TMDs hetero-structures. And the preparation processes of hetero-structures are complicated, which is still a big challenge in terms of repeatability and controllability [20, 21]. In addition, it is difficult to understand and grasp the spectral characteristics of graphene/TMDs hetero-structures, which largely hinder the application of graphene/TMDs hetero-structures in future optoelectronic devices [22].

In this paper, graphene/WS_2_ and graphene/MoS_2_ hetero-structures were composed of three kinds of semiconductor materials with different dielectric constant, band gap width and absorption coefficient. The 2D materials were directly grown on the single crystal graphene film of SiO_2_/Si substrate to form the graphene/TMDs hetero-structures, which can ensure the clean interface and atomic level transition of hetero-structures. The structure of graphene, MoS_2_ and WS_2_ can be analyzed by the AFM, SEM, EDX, Raman and photoluminescence spectroscopy to master the spectral characteristics of graphene/TMDs hetero-structures, which can be used to prepare the high-speed electron mobility transistors (HEMT) and photoelectric detectors [23–25].

## Methods

### Preparation and Movement of Graphene

**Fig. 1:**
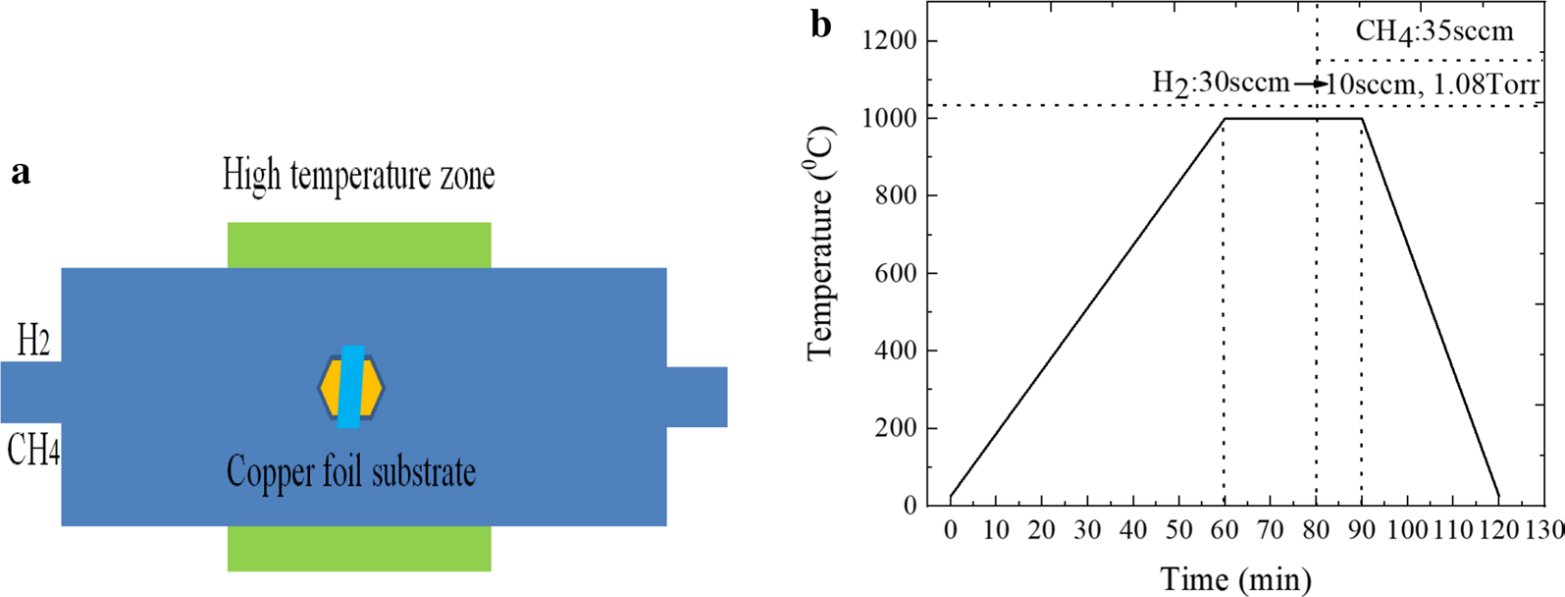
**a** CVD system diagram of graphene growth and **b** the temperature curve during graphene growth

The large-area, high-quality graphene film was prepared by CVD system, which is composed of the tube furnace, gas mixing system and vacuum machine. First, the copper foil with a size of 10 cm × 10 cm was placed into 1 mol/L hydrochloric acid solution for the 3 min ultrasonic cleaning. Then, it was washed with water and ethanol in turn. Subsequently, it was dried by blowing argon gas. Finally, it was inserted in the middle of the quartz tube, and we installed the system and corrected the air pressure [26] (Fig. 1).

As we all know, polycrystalline copper foil would affect the quality of graphene, it is necessary to anneal the copper foil substrate before the growth experiment of graphene. The specific conditions of the annealing processes at stage 1 were the following: the annealing temperature, time and the flow rate of hydrogen (H_2_) gas were 1000 °C, 20 min and 30 sccm, respectively. At this time, the surface of copper foil would form the large area single crystal domain, and H_2_ gas can reduce copper oxide, which can obtain the high-purity copper substrate. The growth temperature remains constant while entering stage 2, the flow rate of H_2_ gas was adjusted to 10 sccm, meanwhile 35 sccm methane (CH_4_) gas was also introduced, the growth time and growth pressure were, respectively, maintained for 10 min and 1.08 Torr, and the growth rate of graphene was approximately 16 μm/s in our CVD experiment, which would ensure the preparation of relatively uniform monolayer graphene film [27, 28]. Finally, the tube furnace was quenched to room temperature at a certain rate, which can avoid damaging the surface of substrate.

The following describes the transfer-specific processes of monolayer graphene material to SiO_2_/Si substrate [29]. First, PMMA solution with a mass fraction of 4% was uniformly spin-coated on the surface of monolayer graphene material with a size of 1 cm × 1 cm, the rotation speed and time were 3000 R/min and 1 min, respectively. Next, the copper foil substrate was etched by the (NH_4_)_2_(SO_4_)_2_ solution with a mass fraction of 3%, and the treatment time was 3–4 h. Then, the PMMA/graphene by the glass slide was rinsed repeatedly in deionized water 2–3 times, and PMMA/graphene was removed to the 50 °C constant temperature table by SiO_2_/Si substrate, which can remove the water vapor between monolayer graphene material and SiO_2_/Si substrate, and the monolayer graphene material can be better attached to SiO_2_/Si substrate. In this step, SiO_2_/Si substrate with a size of 1 × 1 cm^2^ was ultrasonically cleaned with acetone, ethanol and water for 15 min, and the surface of SiO_2_/Si substrate is very clean and uniform, which is conducive to the growth of graphene/TMDs hetero-structures. Finally, PMMA/graphene/SiO_2_/Si was put in acetone solution for 3–4 h to dissolve PMMA and repeatedly wash it with alcohol and deionized water to ensure that monolayer graphene film can be transferred to SiO_2_/Si substrate.

### The Preparation of Graphene/TMDs Hetero-structures

**Fig. 2:**
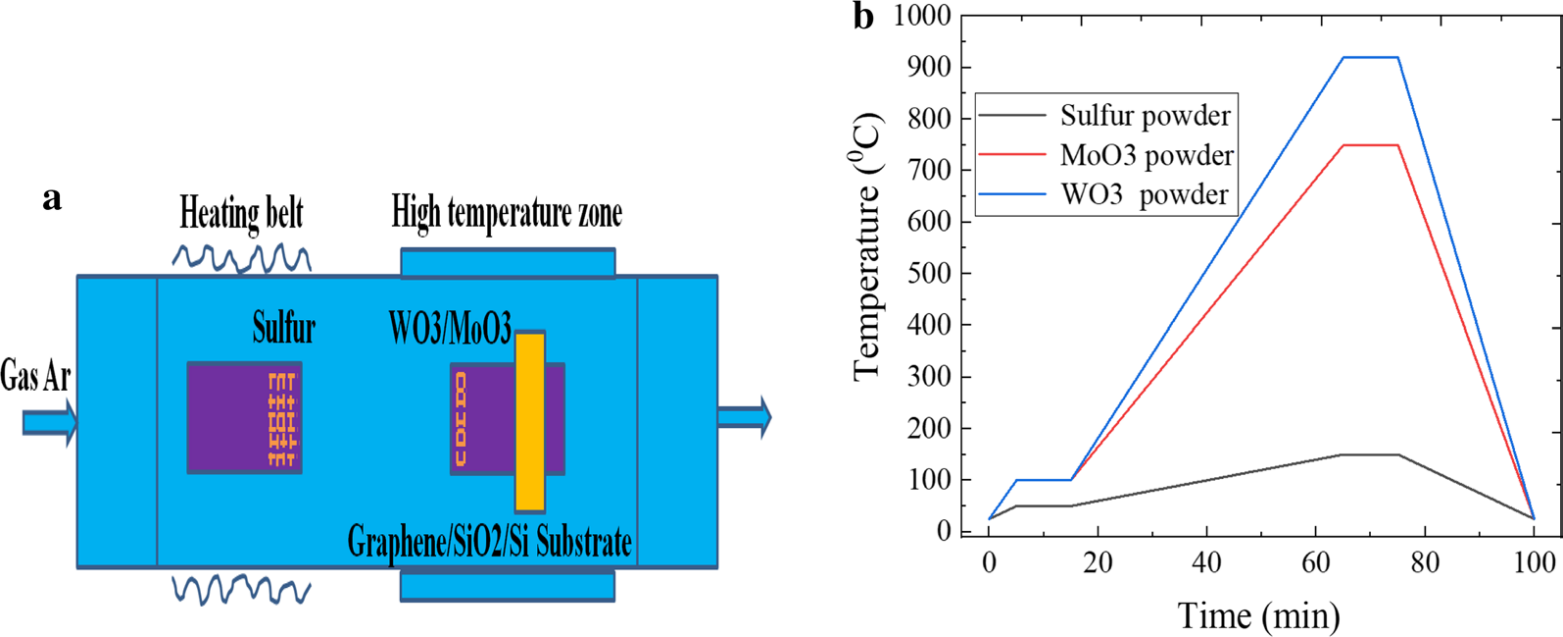
**a** Preparation schematic diagram of graphene/TMDs hetero-structures and **b** the relationship curve between growth temperature and time

In CVD dual temperature zone tube furnace, the graphene/SiO_2_/Si substrate was used for the growth of MoS_2_ and WS_2_ material. The MoO_3_, WO_3_ and sulfur powders were used as the growth molybdenum source, tungsten source and sulfur source, respectively. The high-purity Ar gas was also used to prepare the graphene/MoS_2_ and graphene/WS_2_ hetero-structures, respectively. First, the quartz boat with 100 mg sulfur powder was placed at the upstream of tube furnace. Then, 2 mg MoO_3_ powder (or WO_3_ powder) was filled in another quartz boat, and the graphene/SiO_2_/Si substrate was flipped upside down on MoO_3_ powder (or WO_3_ powder). And then, the quartz boat equipped with the graphene/SiO_2_/Si substrate and MoO_3_ powder (or WO_3_ powder) was inserted into the high temperature area of tube furnace. The heating belt was wound on the quartz tube to heat the sulfur powder, which would ensure that sulfur powder was well controlled and uniformly evaporated, as shown in Fig. 2a. Next, the high-purity Ar gas with a flow rate of 50 sccm was used as carrier gas, the evaporation temperature of sulfur powder was controlled at 150 °C, the growth temperature and growth time of MoS_2_ and WS_2_ were 750 °C, 920 °C and 10 min, respectively. Meanwhile, the first-stage temperature was maintained at 100 °C for 10 min, which can remove the water vapor of tube furnace. The specific temperature change diagram is shown in Fig. 2b. Subsequently, sulfur powder began to sublimate to the sulfur vapor, and the sulfur vapor reaches the high temperature area of tube furnace, which can be driven by Ar gas. It can be fully reacted with MoO_3_ and WO_3_ powder, and the product was deposited on graphene/SiO_2_/Si substrate. Therefore, the growth rate of graphene/TMDs hetero-structures was consistent with that of TMDs materials [30]. After the growth of MoS_2_ and WS_2_ material, the tube furnace was naturally cooled to room temperature, and the color of substrate becomes light yellow.

### The Test Characterization of Graphene/TMDs Hetero-structures

In this paper, the test and characterization methods of graphene/TMDs hetero-structures mainly include the optical microscope (OM), Raman and photoluminescence (PL) spectroscopy, field emission scanning electron microscope (FESEM), energy-dispersive X-ray spectroscopy (EDX) and atomic force microscope (AFM). First, the surface morphology of graphene/TMDs hetero-structures can be observed by optical microscope, SEM and AFM. The layer number of hetero-structures can be judged according to the different contrast of hetero-structure sample. Then, the spectral characteristics of graphene/TMDs hetero-structures were tested and characterized. The growth morphology, growth pattern and growth mechanism of TMDs materials on graphene surface were analyzed and speculated based on the characterization results [31]. Next, Raman spectroscopy has the advantages of quickness, high efficiency and low destructiveness in terms of characterizing 2D materials. It can directly observe the interaction of electron phonons on the sample surface, which has a very wide range of applications in 2D materials. The layer number and crystal quality of 2D materials can be effectively judged by analyzing the characteristic peak position of Raman spectra, the characteristic peak position wave number difference and other characteristics of graphene/TMDs hetero-structures. Finally, the PL spectra were also an important method for characterizing and analyzing 2D materials. When the bulk material is thinned to monolayer material, the band gap width of TMDs material changes from the indirect band gap semiconductor to the direct band gap semiconductor. Meanwhile, the fluorescence effect was significantly enhanced, and there are the obvious characteristic peaks in the PL spectra. However, if the crystal quality of graphene/TMDs hetero-structures was not high, the characteristic peak intensity of PL spectra would be small even if the sample has few layers or monolayer. Therefore, the layer thickness and crystal quality of sample can also be judged by PL spectra. In addition, the distribution, element type and concentration percentage of graphene/TMDs hetero-structures films can be obtained by FESEM and EDX. Meanwhile, the AFM test was also used to grasp the surface cleanliness, roughness and material thickness of hetero-structures film samples.

Both the PL and Raman spectra were collected by the LabRAM HR Evolution high-resolution Raman spectrometer, which was produced by HORIBA Jobin Yvon (French company) [32, 33]. The range of Raman and PL spectra was 300 cm^−1^–3000 cm^−1^ and 550–800 nm, respectively. And the Raman and PL spectra were 10% power and 5% power, respectively. The following were the specific test conditions, spectral resolution ≤ 0.65 cm^−1^; spatial resolution: horizontal ≤ 1 μm, vertical ≤ 2 μm; 532 nm laser, 50× objective lens (beam spot diameter is 1.25 μm, and 100% laser power equivalent to 7500 μw/cm^2^); scan time 15 s, and the cumulative number is 2.

## Results and Discussion

### The Optical Micrograph and Characterization of Graphene/WS_2_ Hetero-structure

**Fig. 3:**
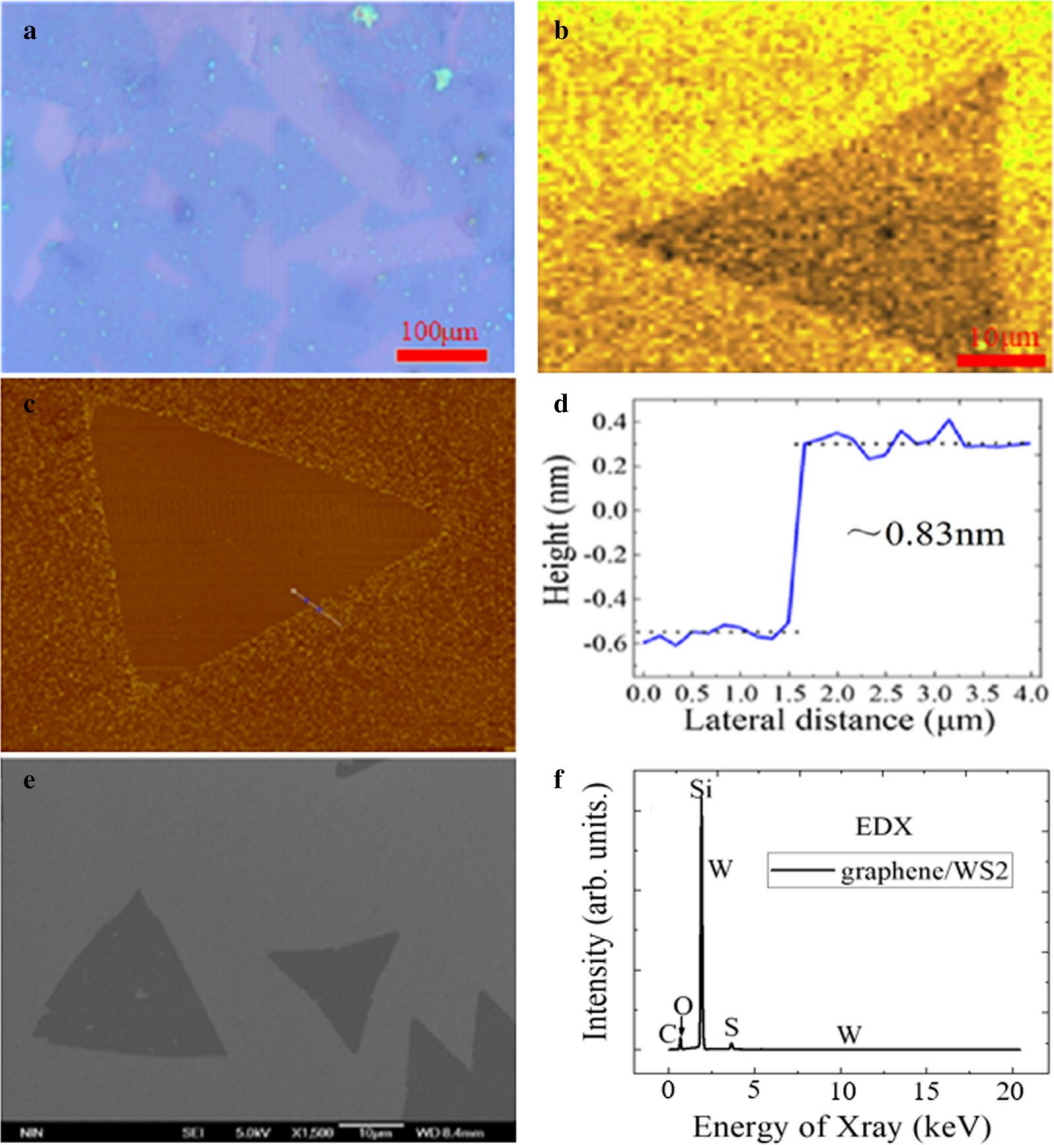
**a** Optical micrograph, **b** mapping image, **c** AFM image, **d** height profile image, **e** FE-SEM image and **f** EDX spectrum of graphene/WS_2_ hetero-structures on SiO_2_/Si substrate

The morphology of hetero-structures can be distinguished by the high-resolution microscope of Raman spectrometer. Figure 3a shows the optical microscope images of graphene/WS_2_ hetero-structure under the different locations of SiO_2_/Si substrate. Since the color of graphene film transferred to SiO_2_/Si substrate was not much different, the graphene film is relatively uniform and complete. The surface of graphene/SiO_2_/Si substrate was clean except for a small amount of particles, which indicates the presence of better quality graphene film. Meanwhile, the nucleation density of WS_2_ became maximum when the gas concentration is sufficient in the growth experiment of WS_2_. And the WS_2_ grown on graphene/SiO_2_/Si substrate was the triangular structure grain with the uniform grain surface and a side length about 120 μm. The shape of WS_2_ was regular and complete, and the thickness was uniform, which is much larger than the size of mechanical peeling sample [34]. In Fig. 3b, since the fluorescence intensity of WS_2_ sample is highly uniformly distributed, the triangular monolayer WS_2_ film has the higher quality and lower defects. It can be seen from Fig. 3c, d that the morphology of WS_2_ film sample is triangle, and the thickness of WS_2_ film is 0.83 nm, which indicates the preparation of monolayer WS_2_ film. In addition, SEM was also used to analyze the morphology of WS_2_ sample film, and the morphology was the regular triangular with uniform thickness, as shown in Fig. 3e. In Fig. 3f, the dock element, sulfur element and carbon element are shown in EDX spectrum, which shows that graphene/WS_2_ hetero-structure material is successfully transferred and prepared.

**Fig. 4:**
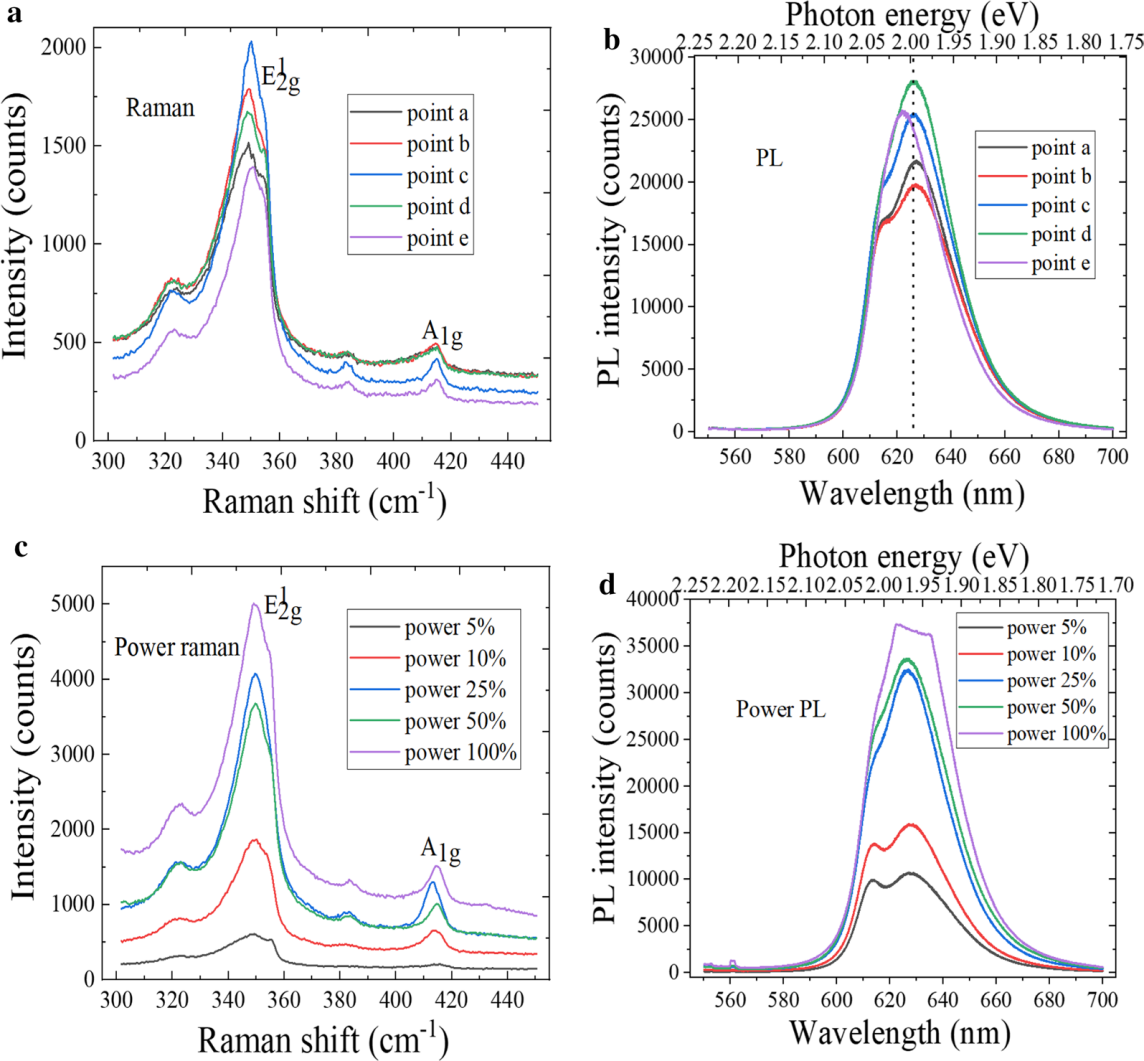
Spectral characterization of WS_2_. **a** Raman spectra at different positions, **b** PL spectra at different positions, **c** power Raman spectra and **d** power PL spectra

The molecular vibration and rotation information of material can be obtained by Raman spectroscopy, which are the fingerprint vibration spectra for identifying the material structure. The layer number and crystal quality of WS_2_ sample can be effectively judged by the characteristic peak position and wave number difference of Raman spectra. Figure 4a shows the Raman spectra of WS_2_ sample at different positions, the E^1^_2g_ and A_1g_ characteristic peaks were located at 350.4 cm^−1^ and 416.1 cm^−1^, respectively. When the bulk WS_2_ changes to monolayer material, E^1^_2g_ and A_1g_ characteristic peaks appear the blue-shifted and red-shifted, respectively. Therefore, the layer number can be judged by the wave numbers difference between two characteristic peaks, and the wavenumber difference was 65.7 cm^−1^, so the triangular WS_2_ grains were monolayer material. In Fig. 4b, the strongest luminescence peak was located at 626 nm, and the corresponding band gap was 1.98 eV, which is consistent with the band gap width of monolayer WS_2_. As we all know, the PL intensity of 2D material is related to the crystal quality and layer number. The 2D material has the fewer defect and layer number, and the luminous intensity is higher, which indicates that the crystal quality is better [35]. The variable power characterization can be performed at the nW level to prevent the laser irradiation from damaging the sample. It can be found by observing Fig. 4c that the peak position of E^1^_2g_ plane vibration mode remains basically unchanged with increase in the excitation power, and the A_1g_ vibration mode between the planes moves to the short wave number direction. This is because the A_1g_ vibration mode has the great relationship with electron concentration, and the increase in electron concentration would lead to the reformation of band gap. As shown in Fig. 4d, the PL spectra intensity of WS_2_ increases with the laser power increases, and there exists the fluorescence quenching phenomenon, which is due to the reformation of band gap and the interlayer interaction of hetero-structures. At the same time, it can also be found that the local temperature of material did nearly not change with increase in the laser power. This is because WS_2_ is the atomic layer-level nanomaterial.

**Fig. 5:**
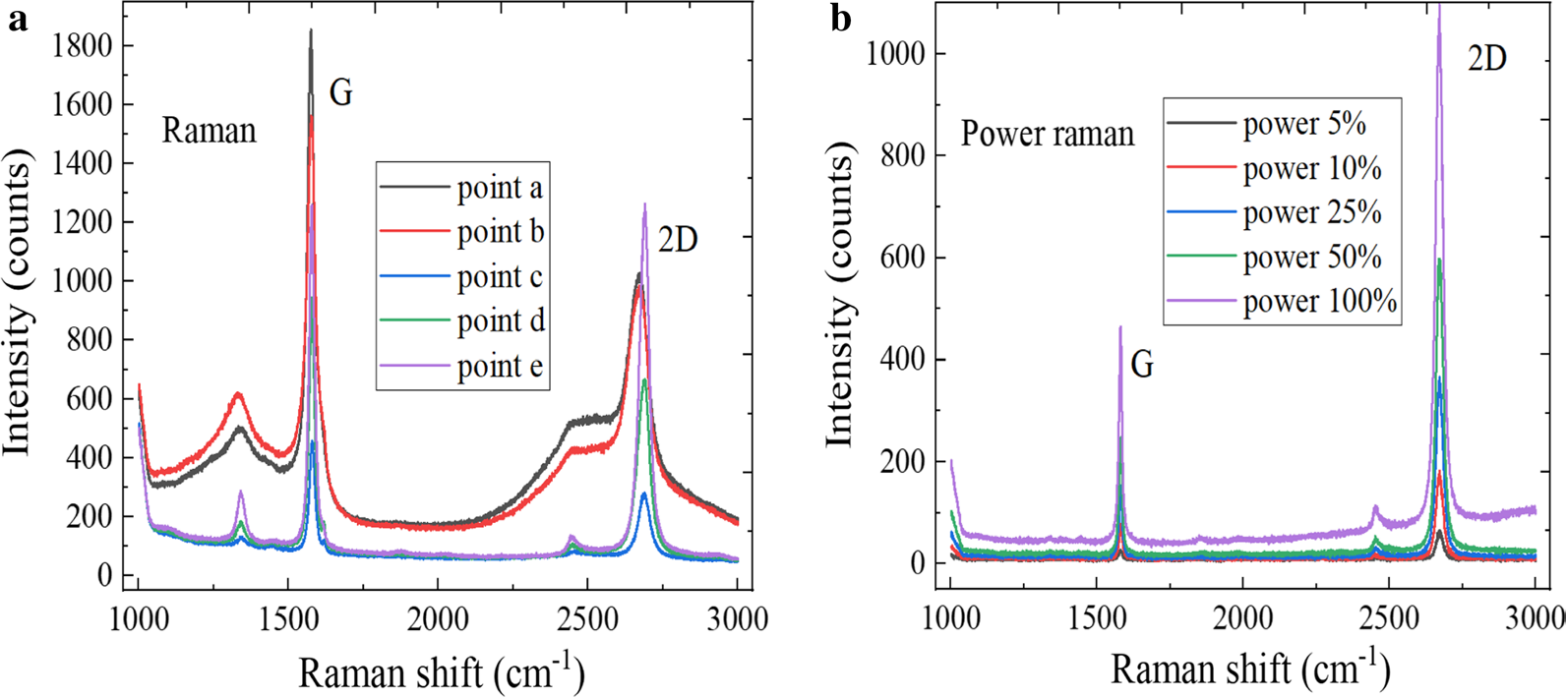
Spectral characterization of graphene. **a** Raman spectra at different positions and **b** power Raman spectra

The layer number characterization and quality information of graphene material can be obtained by Raman spectroscopy. In Fig. 5a, the Raman diffraction spectra of graphene at different positions has the three main characteristic peaks, D peak, G peak and 2D peak, were, respectively, located at 1330 cm^−1^, 1583 cm^−1^ and 2674 cm^−1^. The D peak is related to the disorder of graphene lattice structure, and the position of D peak was blue-shifted when graphene material has more lattice defects, which can reflect the defects and impurity content of crystal. The 2D peak is the two-phonon second-order resonance Raman peak, which can indicate the carbon atoms arrangement of graphene material. Besides, the G peak is caused by the E_2g_ mode of first Brillouin zone center, the peak height increases almost linearly with the layers number of graphene, and the G peak intensity is related to the doping of graphene to a certain extent. The relative ratio of 2D peak and G peak can be used to roughly determine the layers number of graphene, and the ratio of D peak to G peak would decrease when the defect density is increased. The weak D peak appears in the Raman spectra of graphene when the growth of MoS_2_ (or WS_2_) material was completed, which indicates that the graphene domain still maintains the high quality. The 2D peak intensity of exposed graphene area weakened, which is affected by the high-temperature growth process. The full width at half maxima (FWHM) of graphene 2D peak gradually increases with increase in the layers number, and the peak position of 2D peak is blue-shifted, which may be related to the energy band relationship of graphene material. The electronic energy band structure splits with increase in the layers number, and a variety of phonon resonance scattering processes would occur. The exciton peak would be excited by absorbing more energy, which would lead to the blue shift of 2D peak position. The peak intensity of G peak at Point C and E is significantly higher than that of 2D peak. The *I*_2D_/*I*_G_ ratio decreases with increase in the thickness, and the transferred graphene in this experiment was not very uniform, which is within the allowable range. Figure 5b shows the power Raman spectra of monolayer graphene. The G and 2D peak intensity of graphene is gradually increasing with increase in the laser power and temperature, and there is basically no change of the peak position and FWHM. The G peak and 2D peak were, respectively, located at 1581 cm^−1^ and 2672 cm^−1^, and the intensity of two characteristic peaks differs greatly. Due to the interaction change between graphene and underlying SiO_2_, the characteristic peak ratio of *I*_2D_/*I*_G_ is decreased. Meanwhile, there were no D defect peak of Raman spectra, which indicate that the selected graphene region has a high quality and the carbon atoms are highly ordered.

Raman spectroscopy was used to characterize and analyze the graphene/WS_2_ hetero-structure material, and there were two spectra of 300 cm^−1^ ≤ *ω* ≤ 500 cm^−1^ and 1400 cm^−1^ ≤ *ω* ≤ 3000 cm^−1^, which were fitted by the Lorentz function. There were the E^1^_2g_ and A_1g_ modes characteristic peaks of WS_2_ in the range of 300 cm^−1^ ≤ *ω* ≤ 500 cm^−1^. The E^1^_2g_ phonon mode is the in-plane displacement of sulfur and tungsten atoms, while the A_1g_ phonon mode is the out-of-plane displacement of sulfur atoms, the above phonon mode locations and intervals vary with the layers number. The G and 2D peaks of graphene appear in the spectra region of 1400 cm^−1^ ≤ *ω* ≤ 3000 cm^−1^, and the layer number and crystal quality information of graphene can be obtained according to the intensity ratio and the peak position of characteristic peaks.

**Fig. 6:**
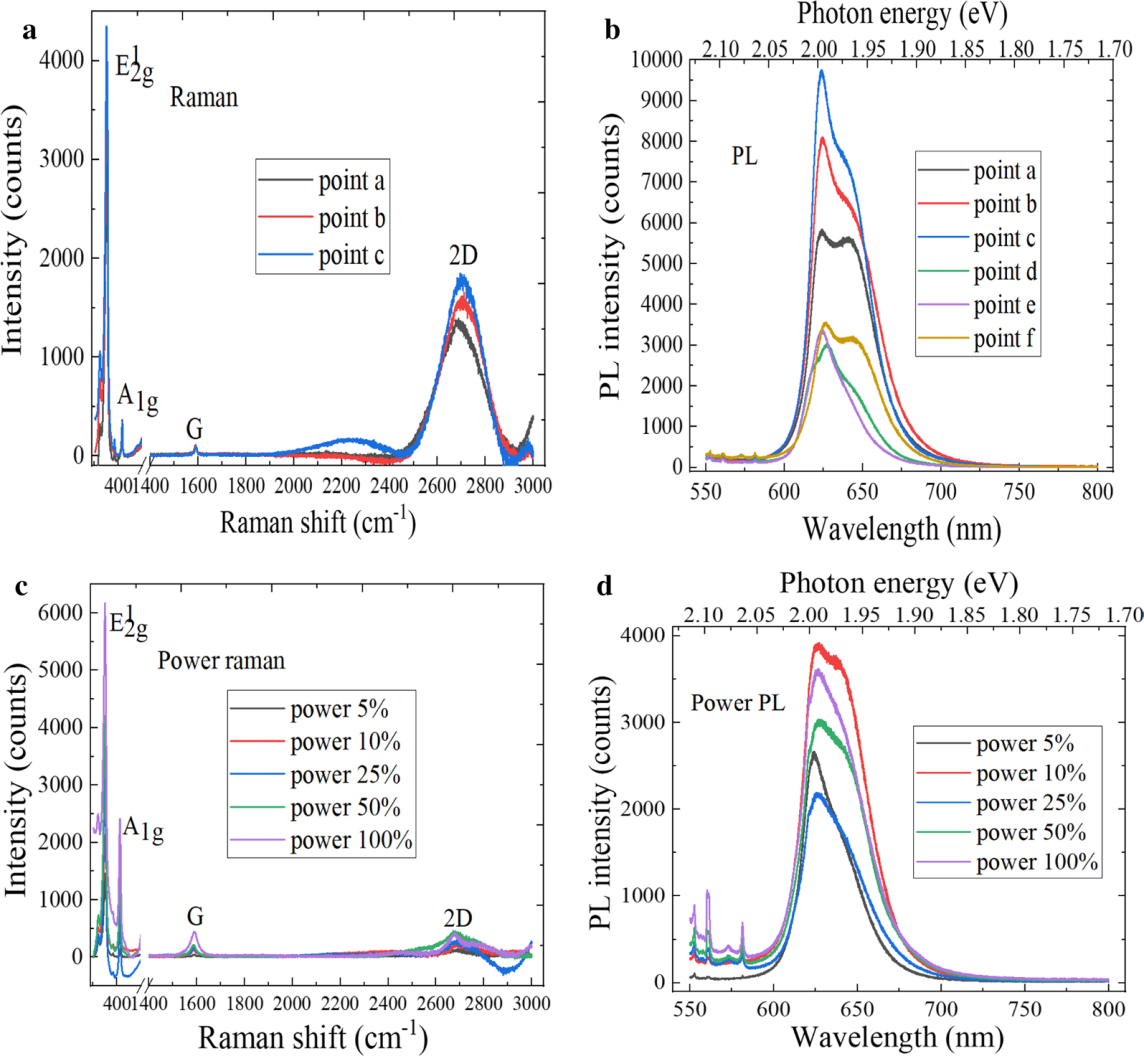
Spectral characterization of graphene/WS_2_ hetero-structure. **a** Raman spectra at different positions; **b** PL spectra at different positions; **c** power Raman spectra; and **d** power PL spectra

The frequency difference of two different Davydov splitting peaks can reflect the interaction magnitude of vdWs hetero-structures. Therefore, the intra-layer vibrating phonon mode frequency of multilayer 2D material also depends on the interlayer coupling and layers number. Figure 6a shows the Raman spectra test characterization of graphene/WS_2_ hetero-structure at different points under the 532 nm laser. It can be found that the intensity of E^1^_2g_ characteristic peak was higher than that of A_1g_ characteristic peak intensity, and the E^1^_2g_ and A_1g_ characteristic peaks were located at 349.3 cm^−1^ and 417.1 cm^−1^, respectively. The Raman spectra 2D and G peaks of graphene/WS_2_ hetero-structure were, respectively, at 1591.5 cm^−1^ and 2680.9 cm^−1^, and the peak position of 2D and G peaks rise compared with pure graphene, which may be related to the effective interlayer coupling of WS_2_ nano-sheets and the strain effect generated by the high temperature heating during CVD growth. The Raman spectra of graphene/WS_2_ hetero-structure material is only the sum of the individual separated WS_2_ and graphene spectra, which can confirm the formation of vdWs heterojunction interface. The PL spectra intensity is related to the crystal quality and layer number. Raman spectroscopy focuses on the influence of hetero-structures formation on the vibration modes, and the electronic band structure of TMDs hetero-structures material can be mainly obtained by PL spectra. Figure 6b shows the PL spectra of graphene/WS_2_ hetero-structure at different points. The strongest luminescence peak was located at 624 nm, and the corresponding band gap was 1.99 eV, which is consistent with the band gap width of monolayer WS_2_. The graphene/WS_2_ hetero-structure material at different positions has the different intensity and shape of PL spectra, and the crystal quality is not very good. Therefore, the preparation processes of hetero-structure need to be further improved. The PL spectra intensity of graphene/WS_2_ hetero-structure is weaker than that of WS_2_. This is because the inter-layer coupling of graphene/WS_2_ hetero-structure changes the exciton fluorescence of hetero-structures region, which would lead to the separation of electron–hole pairs and the reduction in fluorescence. Meanwhile, the peak position shifts when the graphene/WS_2_ hetero-structure is formed, and the transfer of charge can cause the shift of Fermi surface, which can make the free excitons change into the charged excitons. Figure 6c shows the power Raman spectra of graphene/WS_2_ hetero-structure. The in-plane phonon mode E^1^_2g_ characteristic peak and the out-of-plane phonon mode A_1g_ characteristic peak were, respectively, at 356 cm^−1^ and 418 cm^−1^, where the above characteristic peak is increased with increase in the laser power. The peak position and shape of characteristic peak were uniform within single crystal, and the electronic characteristics of WS_2_ on graphene/SiO_2_/Si substrate were uniform. The thickness of WS_2_ sheet can be determined according to the frequency difference between A_1g_ and E^1^_2g_ characteristic peaks, and the average distance was 62 ± 0.2 cm^−1^, which is consistent with the thickness of monolayer WS_2_. Compared to the peak positions of intrinsic graphene, the G peak and 2D peak positions of graphene/WS_2_ hetero-structure from 1578.7 cm^−1^ and 2685.8 cm^−1^ change to 1582.2 cm^−1^ and 2689.5 cm^−1^, respectively. Besides, the intensity of G peak becomes stronger than that of 2D peak with increase in the laser power, and decrease in the *I*_2D_/*I*_G_ ratio, which is caused by the interaction change between graphene and SiO_2_/Si substrate [36, 37]. It can be found by observing Fig. 6d that the PL intensity of graphene/WS_2_ hetero-structure is increased with increase in the laser power**,** the FWHM of PL spectra also increasing, and the shape of PL spectra is changed. The reason is that the test temperature around hetero-structure is increased, and there is also the strong interlayer coupling at the interface of graphene/WS_2_ heterojunction.

**Fig. 7:**
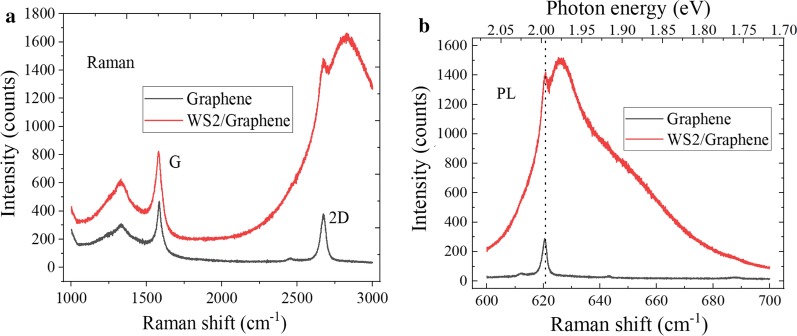
**a** Raman spectra and **b** PL spectra characteristics comparison of graphene before and after WS_2_ growth

The Raman spectra of graphene/WS_2_ hetero-structure were significantly different from that of exposed graphene region, as shown in Fig. 7a. First, the spectral background rises when wavenumber increases, and the background comes from the PL spectra of WS_2_, which confirms the presence of graphene/WS_2_ hetero-structure. Next, WS_2_ material can suppress the 2D characteristic peak intensity of graphene. Finally, both G peak and 2D peak of graphene/WS_2_ hetero-structure shift upward compared with the spectra of bare graphene material. Due to the interlayer coupling between graphene and WS_2_, the 2D peak would also shift up, and the mechanical strain also has the impact on the Raman shift of graphene. The enhancement factor (EF) is the ratio of the maximum peak intensity of graphene/WS_2_ hetero-structure divided by the maximum peak intensity of graphene. The maximum peak intensity of G peak increases from 460 to 830, and the maximum peak intensity of 2D peak increases from 340 to 1460, and the corresponding EF were 1.8 and 4.3, respectively. The D peak signal is significantly enhanced when the graphene/TMDs hetero-structures is formed. Therefore, the *I*_D_/*I*_G_ ratio of monolayer graphene is weaker than that of graphene/WS_2_ hetero-structure. This is because the extrusion of WS_2_ on graphene has the effect on the structure of graphene, which would result in the appearance of a small number of defects. In Fig. 7b, the PL intensity of graphene/WS_2_ hetero-structure is higher than that of bare graphene, which may be related to the effective interlayer coupling and the strain effect. Meanwhile, the maximum intensity of PL spectra is increased from 270 to 1410, and the corresponding EF is 5.23. The intensity enhancement of characteristic peak can be attributed to the coupling of graphene/WS_2_ hetero-structure.

**Fig. 8:**
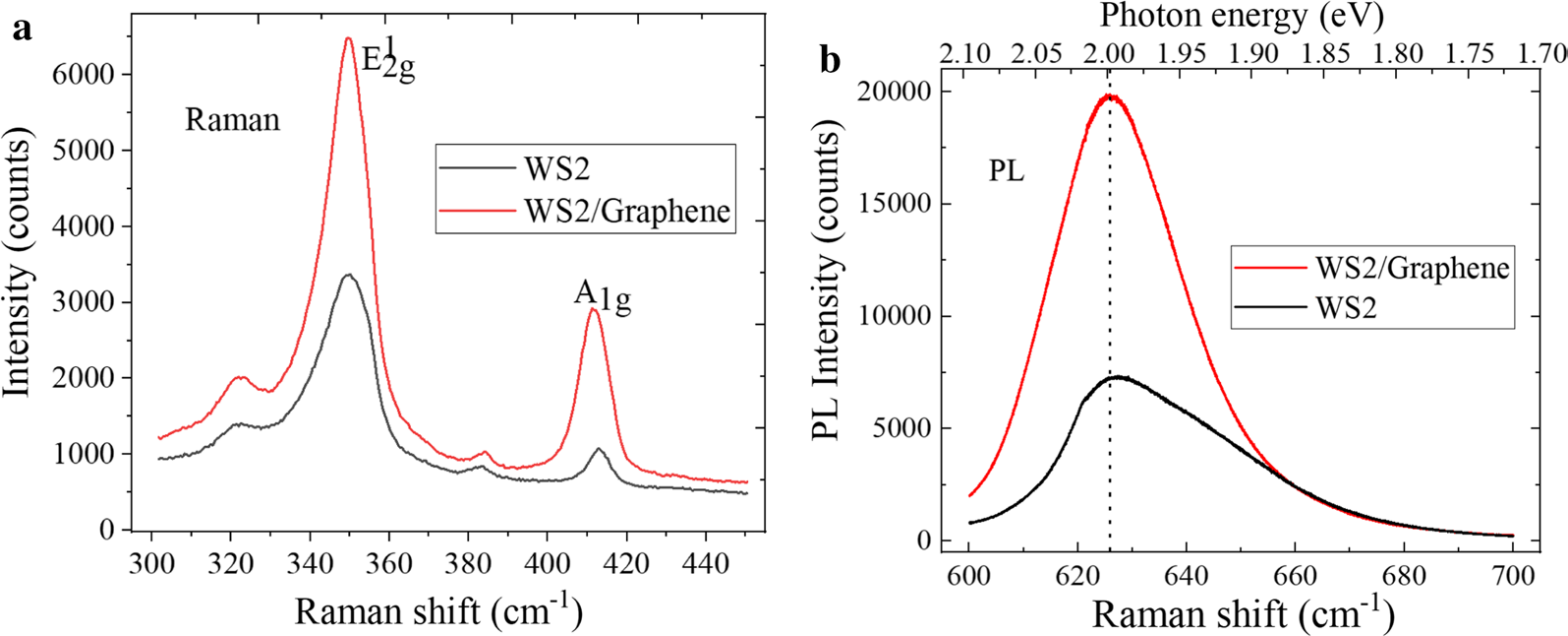
**a** Raman spectra and **b** PL spectra characteristics comparison between WS_2_ and graphene/WS_2_ hetero-structure

Raman spectroscopy can be used to evaluate the crystal quality and film thickness of 2D materials. The Raman spectra comparison of WS_2_ and graphene/WS_2_ hetero-structure is shown in Fig. 8a. Compared to the Raman spectra of WS_2_, the A_1g_ mode characteristic peak position of graphene/WS_2_ hetero-structure was blue-shifted, and the intensity of E^1^_2g_ mode and A_1g_ mode characteristic peaks was higher than those of WS_2_, and the graphene/WS_2_ hetero-structure film has the excellent crystallinity. The reason is that the coupling between layers can be enhanced when the two materials are stacked to form the hetero-structure, which would generate the interlayer interaction forces. The maximum E^1^_2g_ and A_lg_ characteristic peak intensity increases from 3400 and 1100 to 6500 and 2950, respectively. And the enhancement factors (EF) are 1.9 and 2.7, respectively. In addition, monolayer WS_2_ and multilayer WS_2_ are the direct band gap semiconductor and indirect semiconductor materials, respectively. Therefore, the PL spectroscopy can be used to identify the layer number of WS_2_ sample. In Fig. 8b, the above two materials show that the strongest PL emission was around 626 nm, and that the band gap was approximately at 1.98 eV, which is consistent with band gap of the mechanically peeled monolayer WS_2_. The PL intensity of graphene/WS_2_ hetero-structure was stronger than that of monolayer WS_2_. The reasons are the following: First, the work function between graphene and WS_2_ does not match. Second, the internal field was formed. Third, the photoelectrons from WS_2_ can transfer to graphene. Forth, the WS_2_ material retains holes. The maximum intensity of strongest peak increases from 7450 to 19,320, and the EF of PL spectra are 2.6. The increase in peak intensity is due to the coupling between graphene and WS_2_ materials.

### Optical Micrograph and Characterization of Graphene/MoS_2_ Hetero-structure

**Fig. 9:**
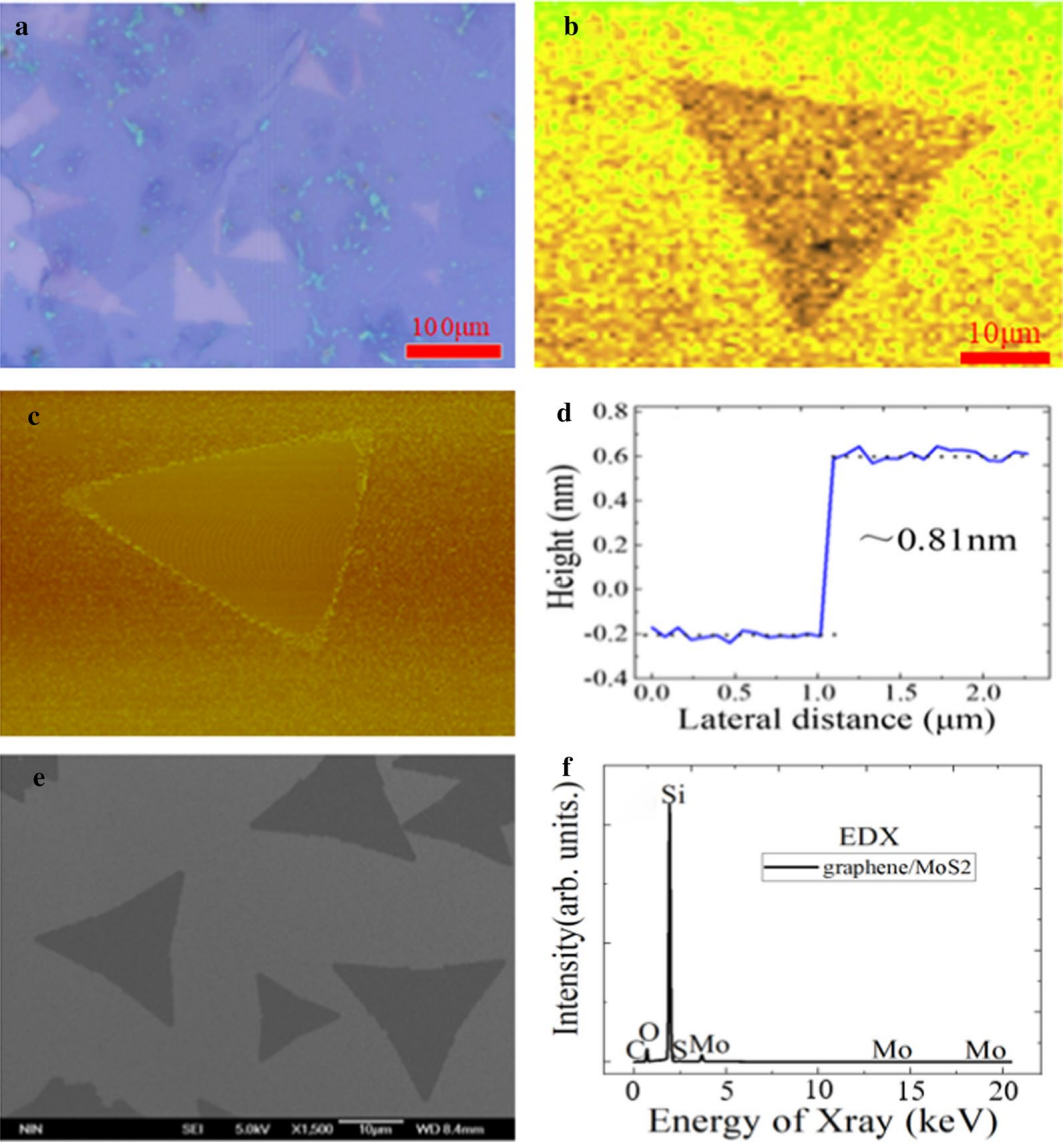
**a** Optical micrograph, **b** mapping image, **c** AFM image, **d** height profile image, **e** FE-SEM image and **f** EDX spectrum of graphene/MoS_2_ hetero-structure on SiO_2_/Si substrate

The optical microscope pictures of graphene/MoS_2_ hetero-structure on SiO_2_/Si substrate are shown in Fig. 9a. We found that the color of the graphene transferred to SiO_2_/Si substrate was not much different from the original one. The surface was relatively clean except for a few particles in some areas. These results indicate that the graphene film is uniformly and completely formed. The MoS_2_ thin film covers graphene/SiO_2_/Si substrate, which can be connected into the continuous graphene thin film across the grain boundaries. The prepared graphene/MoS_2_ hetero-structure was continuous and intact, and the sample surface was relatively clean, which has the good surface uniformity. The local fluorescence intensity distribution is not uniform when there are many defects. Figure 9b shows the in-plane fluorescence intensity distribution of triangular monolayer MoS_2_ film. The crystal lattice of sample has the fewer defects. In Fig. 9c, d, the surface condition of the material is observed by AFM, and the height difference between the edge of the material and the graphene/SiO_2_/Si substrate is measured to judge the material thickness, the thickness of monolayer MoS_2_ material is about 0.81 nm. It can be found by the SEM test result that the morphology of MoS_2_ film sample is the triangular flake, as shown in Fig. 9e. It can be found by observing Fig. 9f that the molybdenum, sulfur and carbon elements are uniformly distributed in the EDX spectrum, which indicates that the graphene/MoS_2_ hetero-structure has been successfully prepared.

**Fig. 10:**
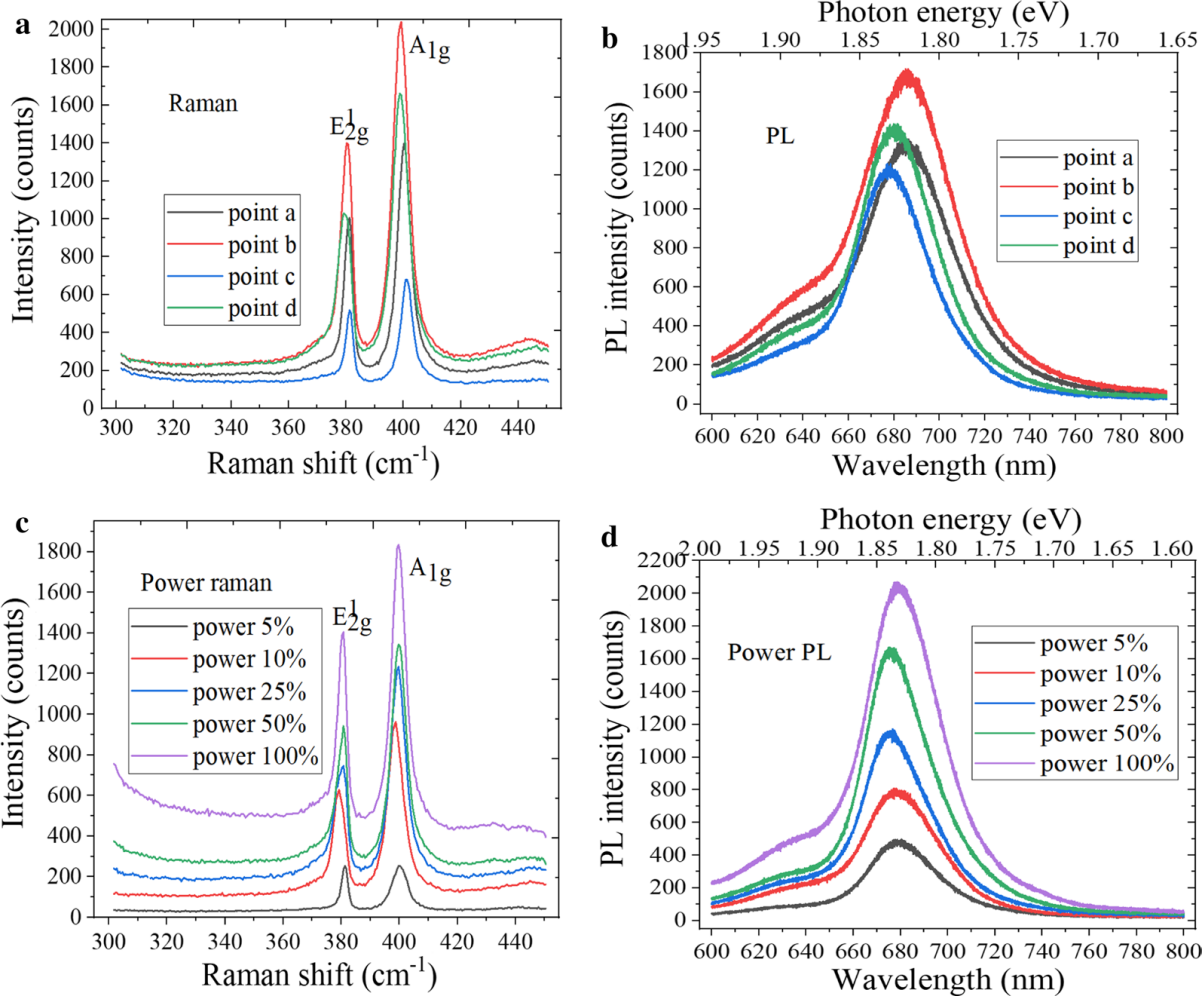
Spectral characteristics characterization of MoS_2_. **a** Raman spectra at different positions, **b** PL spectra at different positions, **c** power Raman spectra and **d** power PL spectra

The interlayer interaction weakens with decrease in the film thickness. The A_1g_ mode characteristic peak is red-shifted, whereas the characteristic peak of E^1^_2g_ mode is blue-shifted. As a result, the frequency distance between A_1g_ and E^1^_2g_ vibration modes becomes smaller, which can be used to identify the thickness of 2D materials. Figure 10a shows the Raman spectra of MoS_2_ at different positions. The characteristic peaks of E^1^_2g_ mode and A_1g_ mode were at 381.2 cm^−1^ and 400.5 cm^−1^, respectively. And the peak spacing was 19.3 cm^−1^, which indicates the presence of monolayer MoS_2_. Due to the Van der Waals force between the layers, the frequencies of two vibration modes moving in the same or opposite directions between adjacent atoms in the layers are slightly different. The PL spectra are used to obtain the light emission characteristics of MoS_2_ film, as shown in Fig. 10b. As we all know, the luminous intensity of monolayer MoS_2_ was much greater than that of multilayer, and the electronic band structure changed from indirect band gap to direct band gap when the layer number of MoS_2_ material changed from multilayer to single layer. Therefore, there was only the strong emission peak of monolayer MoS_2_. In addition, the strongest PL peak was at 678.5 nm, and the corresponding direct band gap was 1.83 eV, which is close to the band gap value of mechanically peeling MoS_2_ film. It can be found by observing Fig. 10c that the characteristic peak intensity of Raman spectra is increased with increase in the laser power and that the peak positions of E^1^_2g_ and A_1g_ mode characteristic peak were blue-shifted. This is because the Raman peak line would have a certain frequency shift with increase in the temperature and laser power. Figure 10d shows the power PL spectra of MoS_2_, the luminous intensity increasing accordingly with increase in the laser power, and the strongest PL peak position was blue-shifted.

**Fig. 11:**
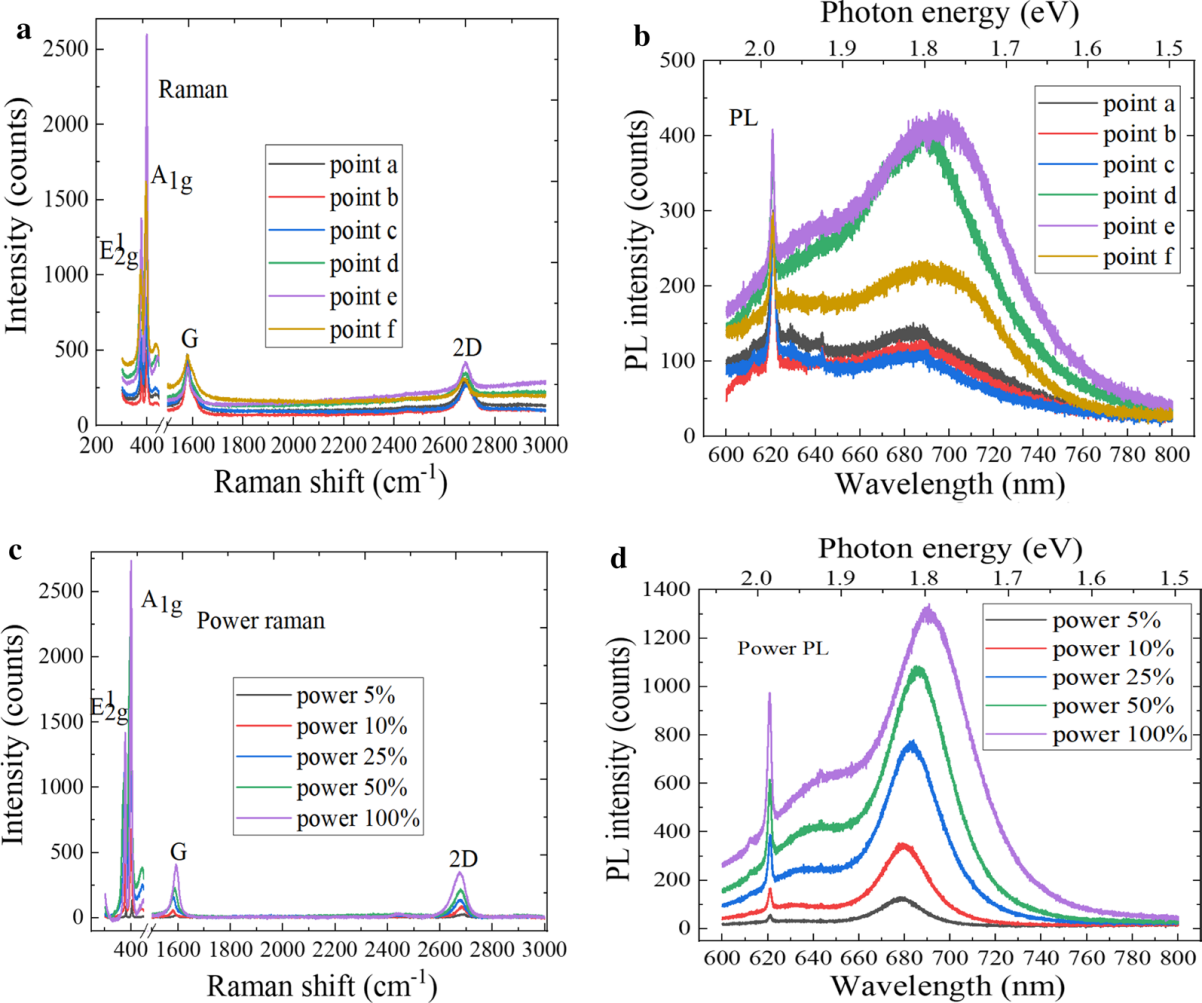
Spectral characteristics of graphene/MoS_2_ hetero-structure. **a** Raman spectra at different positions, **b** PL spectra at different positions, **c** power Raman spectra and **d** power PL spectra

A high-frequency layer vibrating phonon mode of monolayer 2D material would split into the N corresponding high-frequency modes in an N-layer 2D material, which would lead to the Davydov splitting. Figure 11a shows the Raman spectra of graphene/MoS_2_ hetero-structure, and there were the G, 2D peaks of graphene and the E^1^_2g_ and A_1g_ peaks of MoS_2_, which indicates the formation of layered graphene/MoS_2_ hetero-structure material. The E^1^_2g_ and A_1g_ Raman characteristic peaks of MoS_2_ were located at 375.5 cm^−1^ and 394.4 cm^−1^, respectively. And the peak spacing was 18.9 cm^−1^. Compared with intrinsic graphene, the G peak and 2D peak positions of graphene/MoS_2_ hetero-structure shift to large wavenumbers, and G peak and 2D peak move from 1581 and 2672 cm^−1^ to 1587 and 2674 cm^−1^, respectively. In addition, the intensity of G peak is stronger than that of 2D peak. The rise of the 2D and G peaks position is related to the effective interlayer coupling and the strain effect. Compared with the Raman spectra of MoS_2_ material, the spectra of graphene/MoS_2_ hetero-structure material are significantly shifted due to the enhancement of interlayer atomic interaction, and the peak intensity can also be significantly enhanced. It can be found from Fig. 11b that the graphene/MoS_2_ hetero-structure has two absorption peaks at 621 nm and 683 nm and that the corresponding band gaps were 1.99 eV and 1.82 eV according to the conversion relationship between wavelength and electron volt. The luminous intensity of graphene/MoS_2_ hetero-structure was lower than that of intrinsic MoS_2_. The reasons of these phenomena are that the graphene material has the weakening effect on the fluorescence of MoS_2_ material and that the electronic energy band and electronic distribution can be changed due to the interlayer coupling, which can greatly change the PL and Raman spectra.

Figure 11c shows the power Raman spectra of graphene/MoS_2_ hetero-structure, the Raman peaks intensity of G, 2D, *E*^1^_2g_, and A_1g_ increasing with increase in the laser power. The peak position difference between E^1^_2g_ and A_1g_ is gradually enhanced with increase in the layer number of MoS_2_ material. The characteristic peak positions of E^1^_2g_ and A_1g_ were 377.2 cm^−1^ and 396.7 cm^−1^, respectively. And the peak position difference was 19.5 cm^−1^, which can be judged that MoS_2_ material is the monolayer. Meanwhile, the G and 2D peaks of graphene were red-shifted and blue-shifted, respectively. This is because graphene material is doped with MoS_2_. It can be found by observing Fig. 11d that there were two PL peaks of graphene/MoS_2_ hetero-structure. These PL peak corresponding to the compound transition of A and B excitons, wherein the light emission corresponding to the direct band gap exciton recombination was 1.84 eV, whereas the peak corresponding to the indirect band gap exciton recombination was at 2.0 eV. The luminous intensity of strongest peak is increased with increase in the laser power, and the peak position of the strongest PL spectra is red-shifted. This is due to the *p*-type conductivity of the graphene and the change of band structure when graphene and MoS_2_ materials were stacked. In addition, the arrangement of energy bands at the interface allows the electrons from electron-rich MoS_2_ to transfer to *p*-type graphene material.

## Conclusion

Graphene/TMDs-based hetero-structures, where WS_2_ and MoS_2_ were used as TMDs material, were successfully synthesized directly on graphene films by using APCVD. The morphology, spectral characteristics and luminescence law of hetero-structures can be obtained by AFM, SEM, EDX, Raman and PL spectroscopy, and the hetero-structures show the excellent photosensitivity. Compared with intrinsic graphene material, the G and 2D peak positions of graphene/TMDs hetero-structures are the blue-shifted, the intensity of G peak is stronger than that of 2D peak with increase in the laser power and decrease in the *I*_2D_/*I*_G_ ratio. Due to the presence of internal electric field, the photo-generated electron–hole pairs can be effectively separated at the interface of graphene/TMDs hetero-structures, which could greatly improve the light response. This research could effectively guide the preparation process improvement in large-area, high-quality hetero-structures, and it could also pave the way for the application of graphene/TMDs hetero-structures in the optoelectronic devices field.

## Data Availability

The experiment data supporting the conclusion of this manuscript have been given in this manuscript.

## Abbreviations

2D TMDs: Two-dimensional transition-metal dichalcogenides
vdWs: Van der Waals
HEMT: High-speed electron mobility transistors
PMMA: Polymethyl methacrylate
MoS_2_: Molybdenum disulfide
APCVD: Atmospheric pressure chemical vapor deposition
WO_3_: Molybdenum trioxide
OM: Optical microscopy
PL: Photoluminescence
MoO_3_: Molybdenum trioxide
